# Presidential address

**DOI:** 10.4103/0971-9261.59599

**Published:** 2009

**Authors:** V. K. Raina

**Affiliations:** E-mail: rainaveena2@rediffmail.com

Honorable Chief guest Shri Lalji Verma, Vice-Chancellor Prof. Chooramani Gopal, Chairman organizing committee Dr. R.K.Tandon, Organizing Secretary Prof. S. N. Kureel, Honorary Secretary, IAPS, Dr. V. Sripathi, President elect Dr. A.K.Basu, Past Presidents and Presidents and Secretaries of sub chapters, invited guest faculties, delegates, ladies and gentlemen.

I feel honored to be here as the President of our august association on the inaugural function of the 35th annual conference, at this beautiful city of Lucknow famed for its culture and hospitality.

As I stand here before you, I still remember that when I qualified for my Masters in Surgery in 1972, my Professor O.P. Mishra expressed a desire to start pediatric surgery as a superspeciality in Jabalpur Medical College. This dream became a reality in 1974 when one of the Professors of Surgery switched over to start a Pediatric Surgery unit. This was the first center of the superspeciality in Madhya Pradesh. I was sponsored to undergo pediatric surgery training at KEM Hospital, Mumbai, under Dr. R.K.Gandhi and Dr. S.S.Deshmukh. I rejoined the department in Jabalpur in November 1977 as a fully qualified Pediatric Surgeon.

Since then, we have not looked back and now we are among the centers performing high-volume advanced pediatric surgery and currently pediatric minimal access surgery as well. This is not the fortunate story of Jabalpur only, but of most pediatric surgery centers in this country. Today, Pediatric Surgery in India is on the global map and our trainees are easily accepted overseas because of the excellent grasp of the subject and their technical skills.

The holistic efforts of senior pediatric surgeons in India and the enthusiasm of the younger members have given birth to ‘state of the art’ pediatric urology, pediatric colorectal, and pediatric minimal access surgery centers, but today's need of our association is to further expand and develop many more subspecialty areas: for example, pediatric neuroendosurgery, pediatric hepatobiliary surgery, pediatric robotic surgery, and so on. This will enable our post-graduates and fellows to keep pace with the best in the world.

On the same path of progress, over the last 1 year, I noticed that we have had a lot of CMEs, seminars, conferences, and workshops all across the country. I had the opportunity to attend a few of them and was truly overwhelmed with the exchange of ideas and transfer of knowledge that took place. I congratulate the organizers of all these meets all over the country for their painstaking efforts in enabling members from smaller cities and towns to update their knowledge.

All of us need to applaud and support Prof. D. K. Gupta in his efforts to host the first World Congress of Pediatric Surgery (in India) along with the 36th Annual Conference of IAPS in New Delhi in 2010. I would request all the members of IAPS to actively participate in this prestigious meet and put India on the world map of Pediatric Surgery.

The FAPSS – Federation of Associations of Pediatric Surgeons from SAARC – that was formed in 2004 in Jabalpur meets regularly; however, Indian enthusiasm and participation is very limited. Our members are requested to join hands with their brethren in South Asia. Please remember that our future lies in this geographical area.

I congratulate all the office bearers and members of the subchapters for successfully conducting their scientific activities and having excellent rapport with the parent association in all respects. Congratulations!

The fate of the journal lies in the submission of good-quality scientific material, prompt editorship, and adequate finances for publication. In the last year despite some initial difficulties with finance and with the help of a seed grant from IAPS, the journal has been published as per schedule. Congratulations to Dr. K.L.N. Rao and the JIAPS team and please keep up the good work.

My tenure started with a concern to improve the financial status of the association and with a proud initiative of 38 members we were able to collect Rs. 3,46,500. My sincere thanks to all members who contributed to the corpus and I hope this spirit of giving will flourish in future as well. In this continuum, I transfer Rs. 10,000 to the association account for coming financial year. Dr. Subhash Dalal needs special mention for his magnificent contribution of Rs 5 Lacs to the association. From the interest, one publication will be issued annually based on the proceeds of the symposia of IAPS annual conferences. This is for the first time, the IAPS will have its own publication.

The strength of the association is unity, which was proved true again by my past Presidents in terms of guidance and support offered. I am extremely thankful to them!

The organizers of IAPS conference 2009 have made strenuous efforts to make the event memorable. All whispers of appreciations which are being heard goes to their credit. I hope this will be an unforgettable academic feast.

There are a few areas where my effort was not fruitful. The ‘subject of Pediatric Surgery to be taught to undergraduates by Pediatric Surgeons’ was discussed by me in the national Governing council meeting of Association of Surgeons of India in New Delhi. All of our General Surgery colleagues and office bearers agreed on this and we should now put this point forward with Medical Council of India. This will allow the birth of pediatric surgery at many centers of India.

My tenure was made comfortable and successful by none other than Secretary, IAPS, Dr V. Sripathi. His sincerity, discipline, punctuality toward assignments, in spite of his busy schedules, will be a delightful remembrance for me. His promptness in streamlining the directory, newsletters, IAPS-Yahoo group, and so on was the strength of the association for past 2 years. Lastly, may I advise, my younger colleague pediatric surgeons to have respect for knowledge, time, seniors, and learning, for which there is no age bar!

“If someone feels that they have never made a mistake in their life, then it means that they had never tried a new thing in their life.”
Albert Einstein

My undertakings, which could not be fructified, are conveyed to the President Elect Dr. A.K. Basu. Hereafter, Dr A.K. Basu will shoulder the august responsibility and I am sure he will perform even better.

